# HDL Cholesterol and Non-Cardiovascular Disease: A Narrative Review

**DOI:** 10.3390/ijms22094547

**Published:** 2021-04-27

**Authors:** Emilie W. Kjeldsen, Liv T. Nordestgaard, Ruth Frikke-Schmidt

**Affiliations:** 1 Department of Clinical Biochemistry, Copenhagen University Hospital—Rigshospitalet, 2100 Copenhagen, Denmark; emilie.westerlin.kjeldsen@regionh.dk (E.W.K.); liv.tybjaerg.nordestgaard@regionh.dk (L.T.N.); 2 Department of Clinical Medicine, Faculty of Health and Medical Sciences, University of Copenhagen, 2200 Copenhagen, Denmark

**Keywords:** Alzheimer’s disease, blindness, diabetes, drusen, epidemiology, genetics, high-density lipoprotein, infection, mortality

## Abstract

High density lipoprotein (HDL) cholesterol has traditionally been considered the “good cholesterol”, and most of the research regarding HDL cholesterol has for decades revolved around the possible role of HDL in atherosclerosis and its therapeutic potential within atherosclerotic cardiovascular disease. Randomized trials aiming at increasing HDL cholesterol have, however, failed and left questions to what role HDL cholesterol plays in human health and disease. Recent observational studies involving non-cardiovascular diseases have shown that high levels of HDL cholesterol are not necessarily associated with beneficial outcomes as observed for age-related macular degeneration, type II diabetes, dementia, infection, and mortality. In this narrative review, we discuss these interesting associations between HDL cholesterol and non-cardiovascular diseases, covering observational studies, human genetics, and plausible mechanisms.

## 1. Introduction

High density lipoprotein (HDL) cholesterol has traditionally been considered the “good cholesterol”, because high plasma HDL levels are strongly associated with low risk of atherosclerotic cardiovascular disease (ASCVD) [1], and because the particle is involved in reverse cholesterol transport—the flux of cholesterol from the periphery to the liver for fecal excretion [2]. The first large population-based study to show an inverse relationship between circulating HDL cholesterol concentrations and ASCVD was a cross-sectional study from 1976 [3]. In the 1980s, prospective cohort studies confirmed these findings, however the association was attenuated when adjusting for non-HDL cholesterol [4]. The strong inverse relationship between HDL cholesterol levels and ASCVD subsequently led to the hypothesis that clinical trials increasing HDL cholesterol concentrations would reduce risk of ASCVD.

The association between low HDL cholesterol and ASCVD has now firmly been established as non-causal after the publication of the Mendelian randomization studies [5,6,7], genetic consortia data [8,9,10], and several failed HDL cholesterol-increasing trials [11,12,13,14,15,16]. Cholesterol ester transfer protein (CETP) inhibitors, in specific, have either shown adverse effects, no effects, or small beneficial effects on cardiovascular outcomes, the latter possibly explained by the additional reduction in non-HDL cholesterol (cholesterol content in the atherogenic lipoproteins: low-density lipoprotein (LDL) cholesterol, triglyceride-rich lipoproteins, and lipoprotein(a)) [11,12,13,14,15,16]. This non-causal effect of HDL cholesterol is in stark contrast to the well-established causal effect of high levels of LDL cholesterol (“bad” cholesterol) on ASCVD risk, which is robustly supported by decades of evidence from human genetics, animal studies, observational epidemiology, and randomized clinical trials [17,18].

Interestingly, recent studies have shown that high plasma HDL cholesterol levels are associated with disadvantageous outcomes such as cardiovascular disease, infectious diseases, age-related macular degeneration (AMD), and increased mortality [19,20,21,22,23,24,25]. These developments have impelled a reconsideration of the HDL hypothesis and challenged the widely recognized “good cholesterol” label. In this narrative review, we will discuss established and recent findings on HDL cholesterol and its associations with AMD, dementia, type II diabetes, infections, and mortality by summarizing results from observational and human genetic studies and by summarizing plausible mechanisms.

## 2. Age-Related Macular Degeneration

AMD is a disease causing severe visual loss and blindness in the elderly population. AMD is pathologically characterized by drusen, which are deposits that accumulate extracellularly in the area between the retinal pigment epithelium and Bruch’s membrane, as well as by choroidal neovascularization, with vessels disruptively invading the retina. Collectively, this leads to local inflammation and immune reactions [26,27]. Plasma lipids and lipoproteins have been hypothesized to play an important role in the development of AMD, since drusen are composed of at least 40% lipids consisting of mainly esterified cholesterol and phosphatidylcholine [28,29].

### 2.1. Observational Studies

Numerous independent observational studies have explored the relationships between plasma HDL cholesterol levels and risk of AMD in different populations. Studies report either high or low HDL cholesterol levels to be associated with increased risk of AMD, or find no associations [29,30]. Overall, however, most studies point towards the conclusion that high plasma HDL cholesterol levels are related to increased risk of AMD [29,30]. A recent study including 30,953 individuals from the EYE-RISK and European Eye Epidemiology Consortia demonstrated that high plasma HDL cholesterol levels were associated with any type of AMD. Higher HDL cholesterol levels were associated with increased risk of particularly early AMD and drusen [23]. Per 1-mmol/L increase in HDL cholesterol, a 1.21 odds ratio (95% confidence interval 1.14–1.29) for AMD was observed. Interestingly, extra-large HDL particles with higher total lipid and phospholipid content were also reported as seeming to be drivers in the association with AMD [23]. Another recent study, the largest prospective study to date (including 106,703 individuals from the general population) supported these findings. Both high plasma concentrations of apolipoprotein A1 (apoA1) and HDL cholesterol were associated with increased risk of AMD, with the first being a stronger marker of AMD than the HDL cholesterol level [31]. Individuals in the highest versus the lowest quartile of apoA1 and HDL cholesterol levels had hazard ratios for AMD of 1.40 (1.20–1.63) and 1.22 (1.03–1.45), respectively. Both of these two recent studies concomitantly found an inverse relationship between non-HDL cholesterol (the cholesterol content in triglyceride-rich lipoproteins, LDL, and lipoprotein(a)) and AMD risk [23,31]. Interestingly, a recent cross-sectional study has for the first time reported a correlation between high HDL cholesterol and diabetic retinopathy. Larger and prospective studies would, however, be helpful to further investigate the relationship between HDL cholesterol and risk of diabetic retinopathy [32]. A limitation of observational study designs are that they do not provide answers in relation to causality. Genetic studies are, however, able to illuminate potential causal mechanisms by discovering specific genes associated with AMD.

### 2.2. Human Genetics

Early studies examining genes mutated in rare Mendelian forms of AMD have provided insight into the causes of AMD [33]. Variations in the fibulin gene family were demonstrated to be related with AMD, genetic variants in the Stargardt Disease Gene (*ATP-Binding Cassette transporter A4 (ABCA4)*) were more commonly found in AMD cases than in controls, and linkage studies suggested that chromosome 1q25-32, which harbors the factor H gene, was associated with AMD [34,35,36]. However, variation in these genes only explains a small fraction of the total heritability of AMD. In 2005, four separate candidate gene studies examined selected single-nucleotide polymorphisms (SNPs), and found that variation within the complement factor H gene on chromosome 1 represented the most significant predisposition to AMD [37,38,39,40]. Throughout the last decade, genome-wide association studies (GWAS) have succeeded in identifying novel disease susceptibility genes for AMD [41,42,43]. A GWAS is an approach used in genetic research to screen for associations between genetic variants and specific diseases throughout the genome. By genotyping the majority of common SNPs in each individual, every SNP is tested for a difference in allele frequency between the cases and the controls. Importantly, in relation to cholesterol and lipoprotein metabolism, apolipoprotein E (*APOE*) [41], hepatic lipase (*LIPC*) [44], ATP-binding cassette subfamily A member 1 (*ABCA1*) [42], and cholesterol ester transfer protein (*CETP*) [41,45] have been implicated in AMD susceptibility.

Mendelian randomization studies use genetic variants, which are fixed at conception, as instrumental variables to explore the causal relationship between potentially modifiable risk factors and disease in observational data. Mendelian randomization limits the potential biases which are often seen in observational studies, thus minimizing confounding and reverse causation [46]. Through Mendelian randomization studies, a causal relationship between high plasma HDL cholesterol levels and high risk of AMD has been suggested [47,48]. This suggested association was convincing when high HDL cholesterol was caused by genetic variants in *CETP* and *APOE* [23,48,49]. However, for *LIPC*, genetically low HDL cholesterol was associated with increased AMD risk [48]. These findings suggest the existence of a complex relationship between HDL cholesterol concentrations and/or particle size and their role in AMD risk. Han et al. further suggested that genetically predicted high levels of total cholesterol, LDL cholesterol, apolipoprotein B, and non-HDL cholesterol were associated with low AMD risk [47]. It is not known whether these genetic associations are driven by the specific genetic variants associated with high HDL cholesterol and low LDL cholesterol e.g., variants in *CETP* and *APOE*.

### 2.3. Mechanisms

**Figure 1:**
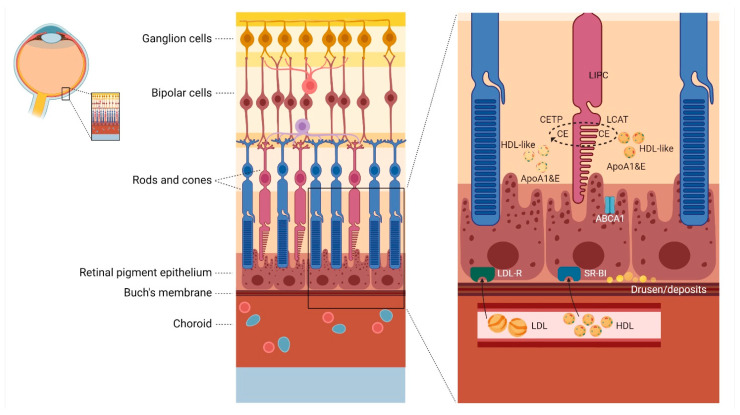
Suggested mechanism of lipid transport in the retina. ABCA1 = ATP-binding cassette transporter A1; ApoA1 = apolipoproten A1; ApoE = apolipoprotein E; CE = cholesterol ester; CETP = cholesterol ester transfer protein; HDL = high-density lipoprotein; LCAT = lecithin:cholesterol acyltransferase; LIPC = hepatic lipase; LDL = low-density lipoprotein; LDL-R = LDL-receptor; SR-BI = scavenger receptor BI. HDL cholesterol, with apoA1 as its major apolipoprotein component, delivers cholesterol to the retina via scavenger receptors, and low-density lipoprotein (LDL) cholesterol delivers cholesterol via members of the LDL receptor family. Cholesterol is subsequently internalized through the RPE and effluxed to the apical side via ABCA1 transporters into the interphotoreceptor matrix to acceptors as apoA1 and apoE. Upon the action of lecithin:cholesterol acyltransferase (LCAT), free cholesterol on the nascent HDL particles are converted into cholesterol esters. HDL is thus transformed into larger and more mature HDL particles by LCAT, while LIPC hydrolyzes phospholipids and thus remodels the HDL particles. Classical apolipoprotein B-containing LDL particles as known from the circulation are absent in the retina, and it has therefore been suggested that CETP has a role in transferring esterified cholesterol between lipoproteins and photoreceptor membranes. Photoreceptor discs are lipid-rich, and HDL may work as a transporter of cholesterol and phospholipids between the RPE and the interphotoreceptor matrix, supporting their synthesis and degradation. The RPE maintains its lipid balance by transporting lipoproteins back to Bruch’s membrane. These lipoproteins have a high abundance of esterified cholesterol and are comprised of both apoA1 and apolipoprotein B, resembling the LDL cholesterol particle except for the content of apoA1. A large amount of esterified cholesterol, perhaps through CETP inhibition, might act as a barrier for lipid transport through an ageing retina, facilitating the formation of deposits or drusen in Bruch’s membrane.

The retinal pigment epithelium (RPE) forms the outer layer of the blood-retina barrier and handles the influx of large quantities of lipids and cholesterol both from the phagocytosis of the outer segments of the photoreceptor and from the endocytosis of lipoproteins from the choriocapillaris [50]. HDL particles have been suggested to be a deliverer of micronutrients essential for the health of the retinal pigment epithelium [28]. HDL cholesterol, with apoA1 as its major apolipoprotein component, delivers cholesterol to the retina via scavenger receptors, and low-density lipoprotein (LDL) cholesterol delivers cholesterol via members of the LDL receptor family (Figure 1) [51,52,53]. Cholesterol is subsequently internalized through the RPE and effluxed to the apical side via ABCA1 transporters into the interphotoreceptor matrix to acceptors as apoA1 and apoE. Upon the action of lecithin:cholesterol acyltransferase (LCAT), free cholesterol on the nascent HDL particles is converted into cholesterol esters. HDL is thus transformed into larger and more mature HDL particles by LCAT, while LIPC hydrolyzes phospholipids and thus remodels the HDL particles (Figure 1) [44,54,55].

Classical apolipoprotein B (apoB)-containing LDL particles, as known from the circulation, are absent in the retina, and it has therefore been suggested that CETP has a role in transferring esterified cholesterol between lipoproteins and the photoreceptor membranes [51]. Photoreceptor discs are lipid-rich, and HDL may work as a transporter of cholesterol and phospholipids between the RPE and the interphotoreceptor matrix, supporting their synthesis and degradation [51]. The RPE maintains its lipid balance by transporting lipoproteins back to Bruch’s membrane. These lipoproteins have a high abundance of esterified cholesterol and are comprised of both apoA1 and apoB, resembling the LDL cholesterol particle except for the content of apoA1 [56]. A large amount of esterified cholesterol, perhaps through CETP inhibition, might act as a barrier for lipid transport through an ageing retina, facilitating the formation of deposits or drusen in Bruch’s membrane. Interestingly, a recent study analyzing apoA1-containing lipoproteins isolated from the Bruch’s membranes of elderly human donor eyes found a unique proteome distinct from HDL, isolated from plasma of the same donor. The most remarkable difference was the higher concentrations of apoB and apoE, which bind to glycosaminoglycans. The authors hypothesized that this interaction promotes lipoprotein deposition onto the glycosaminoglycans of Bruch’s membrane, contributing to RPE dysfunction [57].

*CETP* and *LIPC* genetically control the concentrations of lipid and phospholipid content in HDL particles, and higher concentrations of extra-large HDL particles have been linked to AMD [23]. Phospholipids encompass the outer shell of the lipoprotein and therefore the high phospholipid content of extra-large HDL is likely related to the larger particle size. Inhibition of CETP, however, not only leads to higher levels of HDL cholesterol but also lower levels of non-HDL cholesterol. Therefore, whether it is the circulating high HDL cholesterol, the low non-HDL cholesterol, or local changes in the cholesterol transport and esterification mechanisms in the eye which cause AMD remains to be determined.

## 3. Dementia

Dementia is a devastating neurodegenerative disease and one of the major causes of disability and dependency among older people, currently affecting more than 50 million people worldwide [58]. Alzheimer’s disease is the most common form of dementia, followed by vascular dementia and mixed dementia, the latter displaying elements of both Alzheimer’s disease and vascular dementia [58,59]. Dementia leads to slowly progressing memory loss that advances to deficits in higher intellectual functions and cognitive abilities. These deficits typically affect multiple domains, including language, increasing confusion, personality and behavior changes, and loss of the ability to execute everyday tasks [59]. Dementia often has a subtle onset of symptoms over years and prodromal phases can last for decades [60].

### 3.1. Observational Studies

Cross-sectional studies in general find that low HDL cholesterol levels are associated with increased cognitive impairment [61,62]. Results from prospective cohort studies are conflicting, with both low and high levels of plasma HDL cholesterol being associated with dementia risk [63,64,65,66,67,68]. Reported associations of total and LDL cholesterol are also conflicting. However, some evidence suggests that high levels of total cholesterol in midlife are associated with increased risk of dementia, while high levels in late life are not, as expected due to reverse causation [69]. Reverse causation is when the disease itself causes an alteration in the modifiable trait of interest. As with several modifiable risk factors, late-life measurements often reflect reverse causation, confusing the real impact of risk factors. Many of these studies were indeed conducted in elderly subjects with the simultaneous presence of several risk factors, increasing the possibility of confounding and reverse causation. A recent prospective cohort study from 2019 found that high HDL cholesterol levels were associated with cognitive decline [68]. One study showed that a specific HDL subclass—the concentration of cholesterol esters to total lipids in large HDL—was associated with increased risk of dementia and Alzheimer’s disease [65]. However, this finding not supported by another prospective cohort study [63]. Collectively, results from observational studies within this field are discrepant.

Very high levels of plasma HDL cholesterol have recently been reported to be associated with cardiovascular disease, AMD, infections, and mortality [19,20,21,22,23,24,25]. Whether very high levels of HDL cholesterol are linked to dementia is still unknown, as no large prospective study exists examining plasma levels of HDL cholesterol covering both extreme levels and the risk of dementia and its subtypes. Studies including larger sample sizes, longer follow-up times, and analyses stratified on sex are warranted.

### 3.2. Human Genetics

The apolipoprotein E (*APOE*) gene ε4 allele stands out as an impressive signal for the increased risk of late-onset Alzheimer’s disease, as identified in 1993 [70] and since confirmed worldwide [71]. The role of *APOE* in the peripheral lipid metabolism is well-established, and the common *APOE* ε2/ε3/ε4 polymorphism is associated with all major lipid and lipoprotein traits, including plasma HDL cholesterol levels [72].

ABCA1 is the active transmembrane molecule mediating the transport of cholesterol across cell membranes, with the primary function being to efflux cellular cholesterol onto lipid-poor apoA1 in the circulation, and apoE in the brain. Homozygotes for a loss-of-function mutation in *ABCA1* have Tangier disease, a disease associated with the virtual absence of HDL and apoA1 in the plasma, with cholesterol accumulation in tissues, and with case reports of familial dementia [73]. In 2015, it was clearly demonstrated in a cohort of 91,726 individuals that a low-prevalence loss-of-function mutation in *ABCA1* was significantly associated with low plasma apoE levels, and with high risk of Alzheimer’s disease and cerebrovascular disease [74]. Meta-analyses of GWAS data have now identified more than 70 loci which contribute to the risk of sporadic Alzheimer’s disease [75,76,77,78]. In the most recent and largest GWAS study including 49,589 Alzheimer’s disease cases and 63,575 controls, *ABCA1* was detected as a novel hit associated with Alzheimer’s disease, thus confirming the previous candidate gene study [78].

Using the principles of Mendelian randomization, no causal associations between Alzheimer’s disease and genetically low or high HDL cholesterol levels have been observed [79,80]. However, for some exposures including HDL cholesterol, Mendelian randomization is not always ideal at exploring extreme phenotypes, because conventional Mendelian randomization assumes a linear exposure–outcome association and gives a mean causal effect assumed to be true throughout the scale of the continuous exposure. Furthermore, taking pleiotropy into account will be an important matter for future Mendelian randomization studies within this field.

Whether HDL cholesterol is causally associated with other types of dementia remains to be determined. GWAS studies on vascular dementia and frontotemporal dementia are available. However, they suffer from low power with a limited number of cases, and the subgroups are heterogeneous, limiting the accuracy of gene categorization and pathway analysis [81,82]. When powerful GWAS studies are available for these dementia subtypes, Mendelian randomization analysis should be performed.

### 3.3. Mechanisms

ApoE is the main apolipoprotein in the brain. It is produced locally in the central nervous system (CNS) and incorporated into HDL-like particles that are fundamental for supplying cholesterol to neurons. A cornerstone in Alzheimer’s disease pathology is the accumulation of amyloid-β into amyloid plaques in the brain tissue [83]. Cerebral amyloid angiopathy is characterized by capillary and pericapillary amyloid-β depositions in the brain, resulting in vessel wall fragility. Both Alzheimer’s disease and cerebral amyloid angiopathy are strongly associated with the ε4 allele encoding the apoE4 isoform of apoE, and the clearance process of apoE4-Aβ complexes from brain tissue through capillary endothelial cells is less efficient than that of apoE3-Aβ and apoE2-Aβ [84]. In the brain, ABCA1 lipidates apoE, facilitating the clearance of amyloid-β. Therefore, decreased transendothelial apoE4-Aβ clearance might explain, at least partly, the association between the ε4 allele, cerebral amyloid angiopathy, and Alzheimer’s disease, in individuals with an *ABCA1* loss of function mutation [74].

ApoA1, the main protein component of circulating HDL cholesterol, can enter the brain via transport across the blood brain barrier or the blood cerebrospinal fluid barrier, and be incorporated in the CNS in HDL-like particles [85]. HDL particles may be dysfunctional when plasma HDL cholesterol levels are high, as seen in studies where genetically high HDL cholesterol is due to loss of function variants in scavenger receptor BI, leading to larger and more buoyant HDL particles [24,86]. Perhaps these particles are less efficient at supplying the brain with apoA-I and other important co-factors. Since HDL holoparticle uptake across endothelial structures at the blood-brain barrier is important [87], it is therefore speculated whether high HDL cholesterol levels indicating dysfunctional HDL particles play an important role in dementia pathogenesis.

## 4. Diabetes

Diabetes mellitus is a major public health problem and the worldwide diabetes prevalence is constantly rising, particularly in low- and middle-income countries, posing a substantial health challenge worldwide [88]. In 2014, 422 million people suffered from diabetes globally [88]. Both type I and type II diabetes mellitus are associated with high risks of developing complications such as retinopathy, neuropathy, nephropathy, and atherosclerosis [88]. Type II diabetes, the most common form of the disease, is characterized by hyperglycemia, insulin resistance, and dyslipidemia. A large part of the disease burden from diabetes mellitus arises from different vascular complications that are strongly associated with diabetes mellitus, the pathological hallmarks of which include inflammation, dysregulated angiogenesis, and atherosclerosis [89].

### 4.1. Observational Studies

It is broadly recognized that type II diabetes is often accompanied by dyslipidemia including hypertriglyceridemia and low HDL cholesterol levels in plasma, together with raised apolipoprotein B and the prevalence of small, dense LDLs [90,91]. Observational studies have shown that low HDL cholesterol levels associate significantly with increased risk of type II diabetes [92,93]. As a result of these consistent observational findings, plasma HDL cholesterol elevation has been suggested as a therapeutic option to reduce the risk of type II diabetes and the related vascular complications that come with the diagnosis [94,95,96].

A growing interest in HDL as a potential therapeutic target for type 2 diabetes has emerged [96,97,98]. In clinical trials, post hoc analyses of the cardiovascular outcome have shown that both acute and long-term HDL-raising therapies (four major CETP inhibitors) improved glycemic control and decreased the risk of new-onset diabetes [15,99,100,101]. Whether the effects observed with CETP inhibitors on the reduction of blood glucose concentrations and the risk of diabetes are mediated via their effects on HDL cholesterol is currently unclear, since the glycemic signals could also be due to pleiotropic effects of CETP inhibition. Nevertheless, further studies are needed to clarify the relationship between HDL cholesterol and type II diabetes.

### 4.2. Human Genetics

Large Mendelian randomization studies published between 2015–2016 have shown discrepant results [102,103,104]. A large candidate gene study combining rare and common variants in five key HDL cholesterol genes into a strong genetic instrument, reported that genetically low HDL cholesterol was not associated with type II diabetes in the general population [102]. By contrast, two two-sample Mendelian randomization studies using GWAS data found evidence of an inverse association between genetically determined HDL cholesterol and risk of type II diabetes, indicating a possible causal effect of HDL cholesterol on risk of type II diabetes [103,104]. Despite testing for pleiotropy by applying the Mendelian randomization–Egger method [104], the possibility of pleiotropy outside the pathway cannot be entirely excluded. Pleiotropy is the association of genetic variants with additional phenotypes in alternative disease pathways.

### 4.3. Mechanisms

In type II diabetes patients, abnormalities in glucose metabolism are suggested to be related to impaired HDL function, which is mainly understood as the cholesterol efflux capacity of the HDL particle. A recent study systematically characterized the structure–function relationships of HDL in healthy individuals and patients with type II diabetes or coronary heart disease. They reported that type II diabetes and coronary heart disease are associated with different alterations in HDL: size distribution, protein and lipid composition, and function. Loss of very large HDL and gain of small HDL was observed in patients with type II diabetes [105]. Adipose tissue insulin resistance endorses the release of free fatty acids that are delivered to the liver, consequently driving hepatic triglyceride production [106]. Hepatic insulin resistance then increases the release of very low-density lipoproteins (VLDLs), rich in triglycerides [106]. This subsequent hypertriglyceridemia drives the movement of triglycerides from the VLDLs to HDL particles in exchange for cholesteryl esters, thus lowering HDL cholesterol concentrations [107,108]. Triglyceride-rich HDLs are formed, and they are more susceptible to clearance, which then further lowers not only HDL cholesterol levels, but also the number of HDL particles, suggesting a reduction in HDL functions [109]. However, these mechanisms primarily explain the suggested mechanism in individuals already diagnosed with type II diabetes.

**Figure 2:**
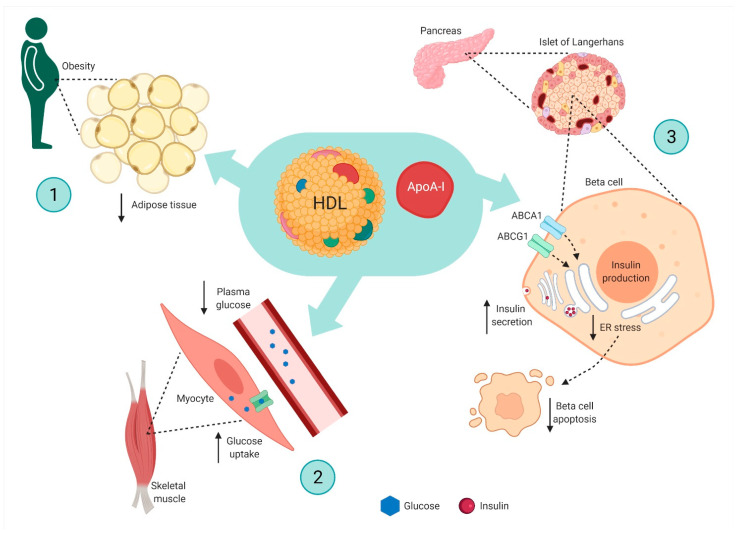
Suggested protective mechanisms of HDL for type II diabetes. ABCA1 = ATP-binding cassette transporter A1; ABCG1 = ATP-binding cassette transporter G1; ApoA1 = apolipoproten A1; ER= endoplasmic reticulum; HDL = high-density cholesterol. HDL may play a causal role in the pathogenesis of type II diabetes through different pathways. (1) The cornerstone of obesity is increased adipose tissue content, and evidence suggests that HDL and apoA1 regulate adipose tissue content. Polymorphisms in the *APOA1* gene have been associated with increased risk of developing obesity in humans, and mice studies have implicated the potential anti-obesity effects of ApoA1. (2) HDL may also play a role in insulin sensitivity and glucose uptake in insulin-sensitive tissues. Studies in humans have shown that reconstituted HDL infusion leads to larger decreases of plasma glucose levels than placebo. Further, ApoA1 transgenic mice have shown lower fasting glucose levels, and a recent study found that a short peptide RH54 derived from ApoA1 induced glucose uptake in cultured muscle myotubes. (3) HDL is suggested to influence pancreatic β-cell function and survival. Deletion of either ABCA1 or ABCG1 in β-cells in mice have resulted in increased intracellular cholesterol levels and hence impaired insulin secretion. Incubation of cultured β-cells with ApoA1 is reported to increase cellular insulin by transcriptional regulation, and ApoA1 has recently been found to prime β-cells to increase insulin secretion in response to high glucose in rats. Lastly, the endoplasmic reticulum is suggested to be a driver in β-cell dysfunction and death. Data from studies in humans and animal models suggest that HDL protects against the development of diabetes through inhibition of endoplasmic reticulum stress and β-cell apoptosis.

HDL is suggested to play a causal role in type II diabetes pathogenesis through different pathways [110]. The cornerstone of obesity is increased adipose tissue content, and it is suggested that HDL and apoA1 regulate adipose tissue content (Figure 2, upper left part). Interestingly, polymorphisms in the *APOA1* gene have been associated with increased risk of developing obesity in humans [111]. Further, mice studies implicate a potential anti-obesity effect of ApoA1. For instance, ApoA1-deficient mice have been shown to have an increased fat content [112] whereas mice overexpressing ApoA1 have decreased gain of white adipose tissue mass when fed with a high-fat diet [113].

HDL may also play a role in insulin sensitivity and glucose uptake in insulin-sensitive tissues (Figure 2, lower left part). This has, for instance, been shown in experiments in patients with type II diabetes where a reconstituted HDL infusion led to larger decreases of plasma glucose levels than placebo, an effect associated with increased insulin secretion [97]. In support of these findings, ApoA1 transgenic mice have shown lower fasting glucose levels and improved glucose tolerance compared with wild-type mice [112,114], and a recent study found that a short peptide RG54 derived from ApoA1 induced glucose uptake in cultured muscle myotubes [115]. In support of this, absence of ApoA1 has led to hyperinsulinemia and hyperglycemia in mice [112]. Another recent study showed that mice treated with HDL or ApoA1 for three consecutive days displayed a reduction in glucagon levels after hypoglycemic stimulus [116]. It is possible that HDLs exert a modulating role in maintaining a normal glucose balance.

HDL has further been suggested to influence pancreatic β-cell function and survival (Figure 2, right part). The deletion of either ABCA1 or ABCG1 in β-cells in mice have resulted in increased intracellular cholesterol levels and thus impaired insulin secretion [117,118,119]. Robust evidence from the general population has not confirmed that these findings in mice translate into increased risk of type II diabetes in humans [120]. Incubation of cultured β-cells with ApoA1 leads to an increase in cellular insulin by transcriptional regulation [121], and ApoA1 was recently reported to prime β-cells to increase insulin secretion in response to high glucose in rats [122]. Endoplasmic stress is suggested to be a driver in β-cell dysfunction and death—the latter a hallmark of diabetes development. Data from studies in animal models and humans suggest that HDL protects from the development of diabetes through the inhibition of endoplasmic reticulum stress and β-cell apoptosis [110,123]. The molecular mechanisms underlying the potential beneficial functions of HDLs in β-cells are largely unknown. Intriguingly, however, a recent study found that a potential protective effect of HDL to reverse endoplasmic stress and β-cell death may involve a complex cascade of the transport, generation, and mobilization of oxysterols for the activation of the hedgehog signaling receptor Smoothened [124].

## 5. Infection

HDL might play an important role in both the innate and adaptive immunity—mechanistically supported by the ability to modulate cholesterol availability in immune cells [125]. One observational study found a U-shaped relationship between HDL cholesterol levels and high risk of infectious diseases, whereas only low LDL cholesterol levels were associated with high risk [25]. In this study, genetically higher HDL cholesterol, via common variants in the genes encoding LIPC and CETP, also showed a trend towards a lower risk of infectious diseases indicating a potential causal role of HDL cholesterol levels [25]. In support of these findings, both observational and genetic data from the UK Biobank found an inverse association between levels of HDL cholesterol and risk of infectious diseases, whereas no genetic evidence for LDL cholesterol and risk was observed. [126] A recent study of clinical genetics and humanized mouse models suggested that inhibiting CETP improves outcome for individuals with sepsis [127]. As previously stated, Mendelian randomization studies determine a linear relationship between the genetic exposure and the outcome. Consequently, the U-shaped relationship found in one of the observational studies [25] would therefore not necessarily be detected in a Mendelian randomization study.

## 6. Mortality

The association between very high HDL cholesterol and high mortality has been reproduced in several independent observational studies including different subpopulations [128]. In general, studies report a U-shaped association between HDL cholesterol levels and mortality in cohorts comprising between 97,166–1,764,986 individuals [19,21,22]. One smaller study exploring the effect of genetically determined HDL cholesterol showed that genetically decreased activity in CETP (and thus genetically elevated HDL cholesterol) was associated with reduced mortality [129]. Decreased CETP activity is, however, additionally related to decreased levels of triglycerides, LDL cholesterol, and non-HDL cholesterol. Currently, there is thus no support for the concept that genetically elevated HDL cholesterol is causally associated with increased risk of mortality, and more studies addressing causality are required. Lastly, clinical trial evidence does not show that HDL cholesterol-raising therapies decrease mortality risk as intended, but either points towards the tendency of increased mortality or no effect [11,15,130].

## 7. Conclusions

**Figure 3:**
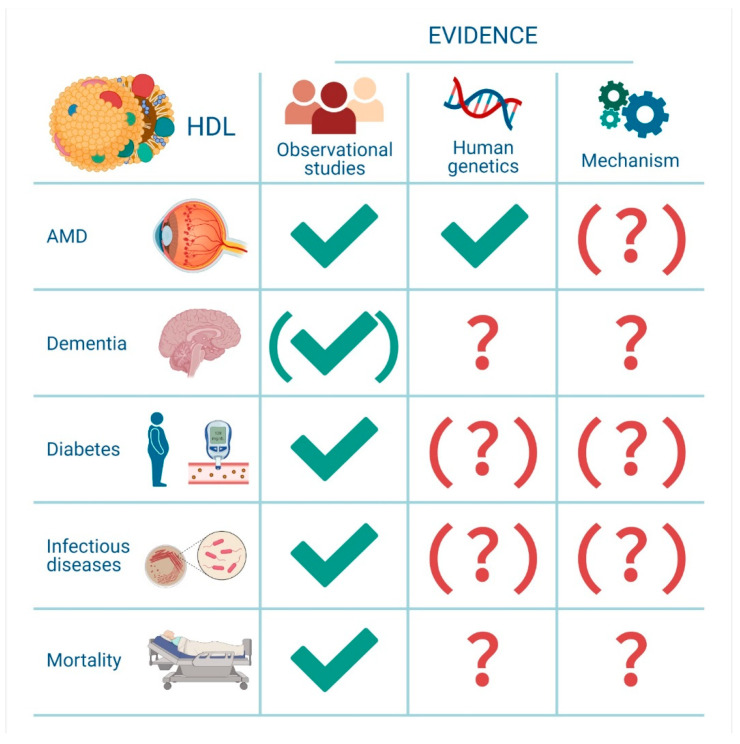
HDL cholesterol and associations with age-related macular degeneration, dementia and type II diabetes. Evidence from observational studies, human genetics and for plausible mechanisms. √ = Substantial evidence; (√) = conflicting evidence; (?) = largely unknown; ? = unknown.

HDL is a complex particle that varies greatly in size and composition, and it is suggested to possess a range of potentially beneficial functions. For seven decades, HDL cholesterol has been perceived as the “good cholesterol.” This narrative review discusses the complex role of HDL and non-cardiovascular diseases, elucidating that high HDL cholesterol levels are not consistently related to a good prognostic outcome (Figure 3).

To summarize, high levels of HDL cholesterol have been established as a potential causal risk factor for AMD via observational and genetic studies. Both low and high levels of HDL cholesterol have been associated with dementia, and further studies are warranted to understand these associations in depth. Whether HDL cholesterol may play a causal role in type II diabetes remains to be determined. There are, however, interesting signals in both experimental and human genetic studies that may indicate a causal relationship between low HDL cholesterol levels and type II diabetes risk. In general, an inverse relationship between HDL cholesterol and infectious diseases is found, also supported by some genetic studies. The association between very high HDL cholesterol and high mortality risk has been reproduced in several observational studies, albeit with no convincing causal support from human genetics and from randomized clinical trials (Figure 3).

Finally, it will be of major importance to determine whether these observational and human genetic findings reflect whether HDL cholesterol concentrations are directly causing the diseases, or whether HDL cholesterol concentrations in plasma mirror a local tissue-specific effect of cholesterol transport or an effect of the size and composition of the HDL cholesterol particle.
